# Observational study on obesity: Insights from middle-aged and elderly college staff in Beijing

**DOI:** 10.1097/MD.0000000000036792

**Published:** 2023-12-29

**Authors:** Chunguo Zhang, Huan Jing, Yan Li, Xiaoling Li, Guojun Xie, Jiaquan Liang

**Affiliations:** a Department of Psychiatry, The Third People’s Hospital of Foshan, Guangdong, China; b Minzu University of China, Beijing, China.

**Keywords:** body mass index, metabolic index, middle-aged and elderly college staff, obesity, physical examination data

## Abstract

Obesity poses a serious global public health challenge, particularly among middle-aged, and elderly college staff. This study aims to explore the associated factors of obesity by analyzing the metabolic indicators of 1756 university staff from Minzu University of China, Beijing. Venous blood samples were collected, and blood metabolic indicators were analyzed. The results indicate that middle-aged faculty members are more susceptible to obesity compared to their younger counterparts. Multiple linear regression analysis revealed that BMI values increase with age (B = 0.074, *P* < .001), uric acid (B = 0.008, *P* < .001), alanine transaminase (B = 0.043, *P* < .001), low-density lipoprotein (B = 1.941, *P* < .001), triglycerides (B = 0.544, *P* < .001), total cholesterol (TC, B = −1.582, *P* < .001), and other factors, while decreasing with the increase of high-density lipoprotein (B = −1.493, *P* < .001). In light of these findings, it is recommended that middle-aged and elderly college staff undergo regular blood indicator checks and enhance weight management to mitigate the risk of obesity and promote their overall health.

## 1. Introduction

In recent years, possibly due to the high intensity of scientific research work and the bad environment of Corona Virus Disease 2019, there had been a sudden death of faculty members in Chinese universities at times, which makes people feel sad.^[1–4]^ Sudden cardiac death (SCD) is one of the major public health problems worldwide, accounting for nearly half of cardiac deaths, and is gaining increasing attention.^[5]^ Studies had shown a dose–response relationship between obesity and SCD, and obesity may lead to increased rates of conditions such as cardiomyopathy, arrhythmias, and coronary artery disease. In conclusion, obesity is now considered the most common nonischemic cause of SCD.^[6]^

The college staff provide excellent talents for the country and play an important role in educating and cultivating future generations. However, the health status of college staff does not seem to receive much attention. Therefore, this study explored the relevant blood test indicators of a university physical examination at the Minzu University of China in 2021, to understand the abnormal body fat of university staff and provide a reference for possible clinical intervention.

## 2. Methods

### 2.1. Participants

The participants were from the Minzu University of China in Beijing. The physical examination data was the employee’s physical examination in 2021, and their age were all over 18 years old. Participants were required to fast for no <8 hours before drawing venous blood, and to complete the blood draw between 7:00–10:00 in the morning with the help of a nurse. The night before blood was drawn, subjects were asked to maintain a normal diet, with the same routine as usual, without strenuous exercise, or drinking alcohol or coffee after dinner.

Exclusion criteria: ① severe and unstable physical diseases, including severe liver and kidney damage, heart failure, diabetes, etc; ② smoking habit (≥1 cigarette per day) or drinking habit (≥1 unit of alcohol per week); 1 unit of alcohol = 480 to 600 mL beer = 350 mL low-alcohol liquor or red wine, yellow rice wine = 50 mL spirits (that is, more than 40 degrees); ③ phobia and other people who cannot cooperate with venous blood drawing.

### 2.2. Assessments

The subjects who met the above conditions, their name, gender, and age were collected through interviews after signing the informed consent form. The height and weight of the subjects were measured using an automatic measuring range meter BSM370 (smitechasia.com), and the BMI (kg/m^2^) was calculated for each subject.

According to the overweight and obesity diagnostic criteria proposed by the China Obesity Working Group (24 kg/m^2^ ≤ BMI < 28 kg/m^2^ is overweight, BMI ≥ 28 kg/m^2^ is obese, and BMI < 24 kg/m^2^ is nonobese).^[7]^

Participants were required to fast for no <8 hours before drawing venous blood, and to complete the blood draw between 7:00–10:00 in the morning with the help of a nurse. The night before blood was drawn, subjects were asked to maintain a normal diet, with the same routine as usual, without strenuous exercise, or drinking alcohol or coffee after dinner. Uric acid (UA), γ-glutamyltransferase (γ-GT), albumin, albumin and globulin ratio (A/G), alanine aminotransferase (ALT), low-density lipoprotein (LDL), high-density lipoprotein (HDL), triglyceride (TG), total cholesterol (TC), blood calcium(CA), blood kalium (K), creative kinase (CK), serum creatinine (SCr), fast blood glucose (FBG), blood urea nitrogen (BUN), lactic dehydrogenase (LDH), basophil, eosinophil, and aspartate aminotransferase (AST) were recorded in the clinical data sheet of the subjects.

### 2.3. Data analyses

Statistical Products and Services Solutions 21 software (SPSS 21, https://www.ibm.com/analytics/spss-statistics-software) was used to analyze the data. Student *t* test and Chi-square test were used to compare differences in subjects’ general demographics and blood metabolic indices. Pearson correlation was used to analyze the relationship between BMI and variables. Next, the obtained *P* values were then corrected by false discovery rate (FDR) correction. Finally, multiple linear regression was used to analyze the effect of blood index components on BMI.

## 3. Result

### 3.1. Comparison of demographic characteristics and metabolic indexes

A total of 1756 participants were included in this study, including young (1363) and middle-aged (393) faculty members of the university. There were no significant differences in K, CK, basophil, and eosinophil (*P* > .05); And there were significant differences in gender, age, BIM, UA, γ-GT, albumin, A/G, ALT, LDL, HDL, TG, TC, CA, SCr, FBG, BUN, LDH, and AST (*P* < .05). The incidence of obesity in young and middle-aged was 3.888% and 8.906%. Overweight was 25.385% and 45.293% (Table 1).

**Table 1:** Comparison of demographic characteristics and test indexes.

	Youth (18–40)	Middle and old age (≥ 40)	t/χ^2^	*P*
n	1363	393	–	–
Gender (male/female)	751/612	287/106	40.570	<.001*
Age (years)	28.800 ± 5.215	43.520 ± 2.664	−50.321	<.001*
BMI (kg/m^2^)	22.678 ± 2.835	24.495 ± 3.232	−10.834	<.001*
Obesity (%)	3.888	8.906	–	–
Over weight (%)	25.385	45.293	–	–
Non obesity (%)	70.727	45.801	–	–
UA	357.420 ± 105.107	335.800 ± 76.969	3.795	<.001*
γ-GT	20.255 ± 14.861	26.865 ± 23.490	−6.724	<.001*
Albumin	47.123 ± 2.815	45.326 ± 2.454	11.463	<.001*
A/G	1.749 ± 0.261	1.598 ± 0.183	10.719	<.001*
ALT	18.644 ± 15.243	23.539 ± 19.327	−5.263	<.001*
LDL	2.914 ± 0.692	3.236 ± 0.739	−8.018	<.001*
HDL	1.443 ± 0.474	1.372 ± 0.343	2.756	.006*
TG	1.027 ± 0.678	1.433 ± 1.179	−8.652	<.001*
TC	4.596 ± 0.854	4.964 ± 0.950	−7.319	<.001*
CA	2.443 ± 0.122	2.344 ± 0.098	14.715	<.001*
K	4.181 ± 0.274	4.165 ± 0.261	1.003	.316
CK	147.558 ± 192.915	142.464 ± 158.417	0.479	.632
SCr	69.952 ± 13.919	72.320 ± 11.168	−3.098	.002*
FBG	5.358 ± 0.408	5.510 ± 0.721	−5.380	<.001*
BUN	4.844 ± 1.175	5.156 ± 1.122	−4.694	<.001*
LDH	156.926 ± 40.088	163.019 ± 33.903	−2.743	.006*
Basophil	0.020 ± 0.015	0.021 ± 0.012	−0.603	.546
Eosinophil	0.135 ± 0.109	0.146 ± 0.121	−1.707	.088
AST	21.010 ± 10.314	23.192 ± 10.507	−3.679	<.001*

A/G = albumin and globulin ratio, ALT = alanine aminotransferase, AST = aspartate aminotransferase, BMI = body mass index, BUN = blood urea nitrogen, CA = blood calcium, CK = creative kinase, FBG = fast blood glucose, HDL = high density lipoprotein, K = blood kalium, LDH = lactic dehydrogenase, LDL = low density lipoprotein, SCr = serum creatinine, TC = total cholesterol, TG = triglyceride, UA = uric acid, value = mean±SD, γ-GT = γ-glutamyltransferase.

* Indicated *P* < .05.

### 3.2. Correlation between BMI and various indexes

The results of Person correlation analysis showed that BMI was positively correlated with age, UA, γ-GT, albumin, A/G, ALT, LDL, TG, TC, CA, K, CK, SCr, FBG, BUN, LDH, basophil, eosinophil, and AST, and negatively correlated with HDL (Table 2).

**Table 2:** Correlation between BMI and various indexes.

BMI								
Age*	*r*	0.189	UA*	r	0.427	γ-GT*	r	0.347
	*P*	<0.001		*P*	<.001		*P*	<.001
Albumin*	r	0.083	A/G*	r	0.066	ALT*	r	.358
	*P*	0.001		*P*	.005		*P*	<.001
LDL*	r	0.235	HDL*	r	− 0.318	TG*	r	.350
	*P*	<0.001		*P*	<.001		*P*	<.001
TC*	r	0.102	CA*	r	0.102	K*	r	.098
	*P*	<0.001		*P*	<0.001		*P*	<.001
CK*	r	0.118	SCr*	r	0.349	FBG*	r	.248
	*P*	<0.001		*P*	<0.001		*P*	<.001
BUN*	r	0.187	LDH*	r	0.258	Basophil*	r	.065
	*P*	<0.001		*P*	<0.001		*P*	.006
Eosinophil*	r	0.073	AST*	r	0.225			
	*P*	0.002		*P*	<.001			

A/G = Albumin and globulin ratio, ALT = alanine aminotransferase, AST = aspartate aminotransferase, BUN = blood urea nitrogen, CA = blood calcium, CK = creative kinase, FBG = fast blood glucose, HDL = high density lipoprotein, K = blood kalium, LDH = lactic dehydrogenase, LDL = low density lipoprotein, SCr = serum creatinine, TC = total cholesterol, TG = triglyceride, UA = uric acid, γ - GT = γ-glutamyltransferase.

* Indicated *P* < .05.

### 3.3. Multiple linear regression analysis of influencing factors of BMI

Taking BMI as a dependent variable (Y) and age, UA, γ-GT, albumin, A/G, ALT, LDL, HDL, TG, TC, CA, K, CK, SCr, FBG, BUN, LDH, basophil, eosinophil, and AST as independent variables (X), gender as a covariate, a stepwise multiple linear regression model (F = 59.515, *P* < .001) was established for analysis. Finally, the elements entering the model were age, UA, γ-GT, ALT, LDL, HDL, TG, TC, CK, SCr, FBG, BUN, LDH, and AST (Table 3). Protective factors and risk factors were 2 relative concepts. The higher the protective factor, the less problem behavior; in contrast, the higher the risk factor, the more problem behavior. The results revealed that HDL was the most significant protective factor for BIM, and the risk factor include age, UA, γ-GT, ALT, HDL, TG, TC, CK, SCr, FBG, BUN, LDH, and AST.

**Table 3:** Multiple linear regression analysis of influencing factors of BMI.

Model	B	Standard error	Standard coefficient	*t*	*P*
(constant)	17.218	1.642	–	10.488	<.001*
Age(year)	0.074	0.010	0.184	7.745	<.001*
UA	0.008	0.001	0.258	9.540	<.001*
γ-GT	0.002	0.005	0.012	0.440	.660
Albumin	−0.052	0.034	−0.049	−1.553	.121
A/G	0.188	0.284	0.016	0.662	.508
ALT	0.043	0.006	0.235	6.686	<.001*
LDL	1.941	0.235	0.459	8.258	<.001*
HDL	−0.360	0.155	−0.054	−2.318	.021*
TG	0.544	0.083	0.151	6.536	<.001*
TC	−1.582	0.196	−0.461	−8.062	<.001*
CA	−0.564	0.738	−0.023	−0.764	.445
K	0.318	0.217	0.029	1.466	.143
CK	0.001	0.000	0.044	2.000	.046*
SCr	0.015	0.006	0.065	2.507	.012*
FBG	0.254	0.122	0.042	2.078	.038*
BUN	0.103	0.052	0.040	1.973	.049*
Basophil	1.239	4.214	0.006	0.294	.769
Eosinophil	−0.299	0.530	−0.011	−0.564	.573
AST	−0.039	0.010	−0.133	−3.968	<.001*

A/G = Albumin and globulin ratio, ALT = alanine aminotransferase, AST = aspartate aminotransferase, BUN = blood urea nitrogen, CA = blood calcium, CK = creative kinase, FBG = fast blood glucose, HDL = high density lipoprotein, K = blood kalium, LDH = lactic dehydrogenase, LDL = low density lipoprotein, SCr = serum creatinine, TC = total cholesterol, TG = triglyceride, UA = uric acid, γ - GT = γ-glutamyltransferase.

* Indicated *P* < .05.

## 4. Discussion

Our study included faculty members who were employed full-time at a university and excluded those who smoked or drank alcohol. Metabolic indicators were included in the analysis to explore the influencing factors of obesity, and to provide a reference for further clinical intervention.

According to a large-scale national epidemiological survey, standardized mean BMI levels rose from 22·7 kg/m^2^ in 2004 to 24·4 kg/m^2^ in 2018, and obesity prevalence from 3·1% to 8·1%, between 2010 and 2018, mean BMI rose by 0·09 kg/m^2^ annually.^[8]^ Our study showed that the incidence of obesity in the youth group and the middle-aged group were 3.888% and 8.906%, respectively, and the middle-aged group was higher than the average level. The college professors are required to teach in the classroom, and they have many other responsibilities, including research activities, organizing seminars and workshops, and supervising the development of trainees and other projects, leading to increased stress with age.^[9]^ And a growing number of studies show that too much stress is one of the reasons for obesity.^[10,11]^ This indicated that the prevalence of obesity increased with the increase of time in the research industry. Therefore, the university staff needed to be paid more attention to weight management in the research progress.^[12]^

The results of this study suggested that age, UA, γ-GT, ALT, LDL, TG, TC, CA, K, CK, SCr, FBG, BUN, LDH, basophil, eosinophil, and AST were positively correlated with BMI, HDL was negatively correlated with BMI, which finally entered the multiple linear regression model.

It is worth noting that CA, K, basophil, and eosinophil entered the multiple linear regression model but the regression was not significant. Basophils and eosinophils are specialized effector cells of the immune system that change with inflammation.^[13]^ Many medical studies had proven obesity was due to chronic inflammation, and basophils and eosinophils play an important role, but the exact relationship was unclear due to the influence of various factors in the body.^[14,15]^ At the same time, studies had shown that basophil and eosinophil count also increased with BMI, but not significantly, which was consistent with the results of this study.^[16]^ Studies had shown that ion channels play an important role in obesity and that K^+^ and Ca^2+^ channels were important regulators of certain functions associated with obesity development, but further studies were needed to explore the link.^[17]^

Through data analysis, age was a risk factor affecting obesity, and with the gradual increase of age, the risk of obesity will also increase. Unlike this study, a previous study showed that men’s BMI decreased with age, while women’s BMI increased with age. This partially overlaps with our findings and may be related to regional differences in participants, and more data are needed to demonstrate this in the future^[18]^

Previous studies had shown that an increased risk of diabetes was significantly associated with a higher UA trajectory, especially in overweight individuals.^[19]^ Human muscle produces many products in the process of metabolism, and SCr was one of them. It was mainly related to the total amount of muscle in the body and did not change with the change in diet. In muscle, SCr was mainly produced by creatine through and irreversible nonenzymatic dehydration reaction, which was transported to the glomerular filtration through blood. In the human body, almost most of the SCr was excreted in the urine.^[20,21]^ An indicator to check whether the kidney function was normal is the detection of SCr. When the SCr in the serum rises, it means that kidney function was impaired. Previous longitudinal studies on diabetes had shown that decreased renal function was positively associated with obesity, which was consistent with the findings of this study.^[22]^

Whether the liver function is damaged was mainly detected by various indicators, among which the detection of γ-GT, AST, and ALT are the most concerning.^[23]^ At the same time, LDH (an important glycolytic enzyme) was also an important indicator of liver function. A large number of studies had proved that obesity and liver damage had a significant positive correlation, and the degree of obesity can be reflected by biochemical indicators (γ-GT, AST, ALT, LDH).^[24,25]^

LDL, TG, FBG, and TC had been unanimously recognized as some of the indicators most related to obesity in routine examinations. With or without diabetes, physical inactivity, and obesity were significantly associated with an increased risk of abnormality on these measures.^[26]^ Therefore, among university staff, regular monitoring of LDL, TG, FBG, and TC values was more important for obesity prevention.

CK was an enzyme that catalyzes energy reactions in muscle cells, and had been shown to modify cardiovascular risk.^[27]^ Studies had also shown that resting plasma CK is independently associated with BMI and indicators of abdominal obesity in a multiethnic population, and CK may be a marker for identifying individuals at risk for obesity.^[28]^ BUN was the nitrogenous product of protein metabolism. Previous studies had shown that the BUN value is positively correlated with BMI, and the reference value of serum BUN was related to age and gender, which varies greatly in the general population, and can be used as one of the criteria for judging obesity.^[29]^

All in all, when the body was at risk of obesity, different indicators will be abnormal, and these indicators had different relationships with people’s liver function, kidney function, heart function, and various lipid metabolism in the body. Abnormalities based on these indicators can make it easier to focus on obesity in people. The university staff was the pillars of the country, and their dyslipidemia was also relatively common, their health situation is worthy of our attention.

Unfortunately, our study did not include all physical examinations, such as excluding those with smoking and drinking habits, which may cause some data biases. The sample source of this study only comes from the Minzu University of China, and the dietary habits belong to northern China, so the follow-up study needs further analysis. The intrinsic link between obesity and different indicators still needs to be further explored, therefore, caution should be exercised in generalizing follow-up conclusions.

In conclusion, the university staff are the wind vane of society and bear the responsibility of educating people, and the development of society needs them. As they grow older, metabolism-related indicators in the body are more likely to be abnormal. They should pay more attention to weight management and health testing, and their physical health deserves attention.

## Author contributions

**Conceptualization:** Jia-Quan Liang.

**Data curation:** Chunguo Zhang, Huan Jing, Yan Li, Jia-Quan Liang.

**Formal analysis:** Huan Jing, Yan Li, Jia-Quan Liang.

**Funding acquisition:** Xiaoling Li, Guojun Xie, Jia-Quan Liang.

**Investigation:** Xiaoling Li.

**Methodology:** Chunguo Zhang, Huan Jing, Yan Li, Jia-Quan Liang.

**Project administration:** Xiaoling Li, Guojun Xie, Jia-Quan Liang.

**Resources:** Xiaoling Li, Guojun Xie.

**Software:** Jia-Quan Liang.

**Supervision:** Guojun Xie, Jia-Quan Liang.

**Writing – original draft:** Chunguo Zhang, Huan Jing.

**Writing – review & editing:** Chunguo Zhang, Huan Jing.
